# A homozygous frameshift variant in the *CILK1* gene causes cranioectodermal dysplasia

**DOI:** 10.1038/s41431-025-01902-0

**Published:** 2025-07-04

**Authors:** Abdullah Sezer, Sukru S. Oner, Hanife Saat, Merve G. Turan, Tulin Gungor, Sebiha Cevik, Asli Erol, Ferhan Yenisert, Kerem Catalbas, Derya Hazal Ozbakir, Sinem Kocagil, Oğuz Cilingir, Mehmet Ali Ergun, Oktay I. Kaplan

**Affiliations:** 1 Department of Medical Genetics, Ankara Etlik City Hospital, Ankara, Türkiye; 2 Department of Medical Genetics, Dr. Sami Ulus Maternity and Children’s Health and Disease Training and Research Hospital, Ankara, Türkiye; 3 Department of Medical Pharmacology, Goztepe Prof. Dr. Suleyman Yalcin City Hospital, Istanbul, Türkiye; 4 Science and Advanced Technologies Research Center (BILTAM), Istanbul Medeniyet University, Istanbul, Türkiye; 5 Department of Medical Genetics, Diskapi Yildirim Beyazit Training and Research Hospital, Ankara, Türkiye; 6 Rare Disease Laboratory, School of Life and Natural Sciences, Abdullah Gul University, Kayseri, Türkiye; 7 Division of Pediatric Nephrology, Department of Pediatrics, Ankara Etlik City Hospital, Ankara, Türkiye; 8 Department of Medical Genetics, Faculty of Medicine, Eskisehir Osmangazi University, Eskisehir, Türkiye; 9 Department of Medical Genetics, Faculty of Medicine, Gazi University, Ankara, Türkiye

**Keywords:** Paediatric kidney disease, Genetics research, Disease genetics, Liver fibrosis, Disease genetics

## Abstract

Cranioectodermal dysplasia (CED) is a ciliopathy characterized by skeletal and ectodermal abnormalities, renal failure, and liver fibrosis. Pathogenic variants in genes that encode the intraflagellar transport (IFT) complex components, particularly IFT-A, are responsible for approximately two-thirds of the CED cases. However, the cause of the remaining cases remains unknown. Ciliogenesis-associated kinase 1 (CILK1) is a highly conserved ciliary serine/threonine kinase with an N-terminal catalytic domain responsible for kinase activity and a C-terminal non-catalytic domain that interacts with the IFT-B complex. Biallelic variants in the catalytic domain are associated with lethal skeletal dysplasia, endocrine cerebroosteodysplasia, and short-rib polydactyly syndrome. No human disease has been linked to biallelic variants in the non-catalytic domain. We present a homozygous frameshift variant in the *CILK1* gene that affects the distal part of the non-catalytic domain, causing CED in five patients from two pedigrees. All the patients survived into childhood and had disproportionately short stature, skeletal abnormalities, ectodermal dysplasia, renal issues, and liver complications. Functional data from patient-derived cells and the *C. elegans* model indicate that the variant reduces cilia number, increases cilia length, and disrupts the localization of IFT components. In contrast, the ciliary localization of *CILK1* bearing the variant itself remains unaffected. Notably, we rescued the majority of these abnormalities by reintroducing *CILK1* into patient-derived cells. Finally, our study describes *CILK1* as a novel causal gene and the first non-IFT protein-encoding gene in the etiology of CED, thus expanding the known genotypic, mechanistic, and phenotypic spectrum of CED.

## Introduction

A unique group of ciliopathies, referred to as skeletal ciliopathies, exhibits overlapping skeletal symptoms, such as short stature, polydactyly, limb shortening, and thoracic narrowing. Cranioectodermal dysplasia (CED) stands out as a distinct subtype characterized by dolichocephaly, skeletal anomalies, ectodermal dysplasia, growth retardation, joint laxity, and unique facial features. Most individuals with CED develop nephronophthisis during infancy or childhood, often progressing to end-stage renal disease, leading to morbidity and mortality. Hepatic fibrosis, retinal dystrophy, and brain imaging findings, such as enlarged extracerebral fluid spaces, can also be observed in the disease. Dolichocephaly and prominent ectodermal features distinguish CED from other ciliopathies. Clinical diagnosis of CED can be based on characteristic findings and radiographic features [1, 2].

Intraflagellar transport (IFT) is a ciliary transport system crucial for cilia formation. It consists of two subcomplexes: the IFT-A complex, which is responsible for retrograde transport and plays a role in ciliary entry and membrane-associated transport; and the IFT-B complex, which mediates anterograde transport. Pathogenic variants in genes encoding the components of both IFT-A and IFT-B are found in nearly all skeletal ciliopathies. CED is particularly associated with pathogenic variants in genes encoding IFT-A complex subunits [3, 4]. While earlier studies reported cilia shortening, reduced ciliation, and ciliary tip bulging due to impaired retrograde transport and accumulation of anterograde cargo [2, 5, 6], recent evidence suggests that CED-associated variants primarily impair ciliary protein trafficking rather than ciliogenesis [7, 8].

Ciliogenesis-associated kinase 1 (CILK1), also known as intestinal cell kinase (ICK), is a highly conserved serine/threonine-protein kinase belonging to the RCK family [9–16]. It contains an N-terminal catalytic domain with kinase activity and a C-terminal non-catalytic domain that mediates interaction with the IFT-B complex [17]. In humans, biallelic pathogenic variants in the catalytic domain of CILK1 have been linked to perinatal-lethal skeletal ciliopathies, including endocrine-cerebro-osteodysplasia (ECO, OMIM #612651) [18, 19] and short-rib-polydactyly syndrome (SRPS) [15]. Moreover, some heterozygous variants of this gene have been reported to cause susceptibility to juvenile myoclonic epilepsy-10 (EJM10, OMIM #617924) [20]. Functional studies across multiple organisms—including *C. elegans*, *Chlamydomonas*, *Tetrahymena*, *Leishmania*, mice, and humans—have shown that CILK1 homologs regulate cilia length and signaling pathways such as Sonic Hedgehog (SHH) [9–16]. CILK1-deficient mouse models display neonatal lethality, skeletal and brain abnormalities, ciliogenesis defects, elongated cilia, and impaired SHH signaling, recapitulating several features of ECO and SRPS [13, 21].

Although the exact prevalence of CED remains unknown, fewer than 100 cases have been reported. Mutations in six IFT genes (*IFT43*, *IFT52*, *IFT122*, *IFT140*, *WDR19*/*IFT144*, and *WDR35*/*IFT121*) have been identified in 66% of patients with CED, but the remaining 34% of patients are still unsolved genetically [1, 2, 5, 6, 22, 23]. Here, we report a homozygous frameshift variant, c.1664_1665delAT: p.(Tyr555Cysfs*48), in *CILK1* in five CED patients from two families. Functional investigations in patient-derived fibroblasts revealed hallmark ciliary abnormalities, including elongated cilia and reduced ciliation, which were rescued by wild-type *CILK1* expression. Additionally, the localization of IFT-B components such as IFT88 and IFT70 was disrupted, while ciliary membrane and basal body markers remained unaffected. Our findings provide the first evidence linking *CILK1* to CED and expand the molecular spectrum of this skeletal ciliopathy, offering new insights for diagnosis and future therapeutic strategies.

## Material and methods

### Recruitment of patients and ethics approval

The subjects were identified according to the authors’ clinical practice. This study was approved by the Institutional Ethics Committee on Human Research at Gazi University, Ankara (approval number#24-27.12.2021). Before participating in the study, all participants or their legal guardians provided informed consent. The methods used in this study complied with the ethical guidelines of the relevant committees on human experimentation. The responsible referring physicians at each local site used standard forms to obtain permission to include their anonymized medical data, including images, in this cohort.

### Genetic analysis

Clinical exome sequencing (ES) was performed on Patients 1–4, and whole ES was performed on Patient 5 using genomic DNA from peripheral blood lymphocytes. Sanger sequencing confirmed the detected variants and screened available parents and relatives. Homozygosity mapping using exome-wide SNPs from ES data was performed for all patients, focusing on regions >3 Mb and recorded in Microsoft Excel. For haplotype analysis, SNP genotypes were examined using NGS data files (VCF and BAM) for all patients, following Taşdelen et al. [24]. Genomic coordinates follow the hg19/GRCh37 genome version, and variants were described using the NM_014920.5 transcript of the *CILK1* gene. Gene names complied with the HUGO Gene Nomenclature Committee (HGNC) recommendations.

### Materials

Total RNA Isolation kit (T2010S), cDNA synthesis kit (E6300S), and restriction digest enzymes were purchased from New England Biolabs. The Neon electroporation system (MPK10096) and cell culture materials such as Amphotericin B (Thermo Fisher J67049.AD), DMEM (Gibco, 21068028), L-Glutamine (Gibco, 25030081), Sodium Pyruvate (Gibco, 11360070), penicillin-streptomycin (Gibco, 10378016), and FBS (Gibco, 10270-106) were obtained from Invitrogen. Antibodies were purchased as follows: rabbit acetylated tubulin (CST-5335), mouse acetylated tubulin (Merck- T7451), polyglutamylated tubulin (Adipogen- GT335), IFT88 (Proteintech- 13967), ARL13B (Proteintech- 17711), CEP164 (Proteintech- 22227), and IFT70 (Proteintech- 25352).

### Cell culture

Under local anesthesia, a 4 mm punch skin biopsy was obtained from both Patient 1 and an age-matched healthy control. The biopsies were sliced into pieces smaller than 0.5 mm using sterile scalpel blades and placed in a 6-well plate. The biopsy pieces were incubated with a regular medium containing 2.5 μg/ml Amphotericin B in a cell culture humidified incubator. The fibroblasts were grown under these culture conditions, and the medium was changed twice a week for approximately one month until a sufficient amount of fibroblast outgrowth was present for further cell passage. When enough cells were in the plates, all the cells were split into new flasks. The cells were then stored in liquid nitrogen. Both patient and control human fibroblasts were maintained in DMEM supplemented with 10% fetal bovine serum, 2 mM glutamine, 100 units/ml penicillin, and 100 mg/ml streptomycin. Cells were grown in a humidified incubator with 5% CO_2_ at 37 °C [25].

### Plasmids cloning

Total RNA was extracted from the RPE-1 cells, followed by cDNA synthesis. The *CILK1* WT gene was amplified by PCR using the following primers to generate specific constructs:

CILK1_forward (XhoI) cggCTCGAGACCatgaatagatacacaacaatcaggcagc

CILK1_reverse (HindIII) gacAAGCTTtcgccgagatgcgtacttgg

After restriction digestion, the PCR product was cloned into the pEYFP-N1 vector. The cloned products were sequenced by Macrogen (the Netherlands), and the Accelrys gene program confirmed the *CILK1* sequence (NM_014920).

To obtain CILK1-expressing stable cell lines, we used the pSBbi-Hgy plasmid (Addgene #60524), a Sleeping Beauty transposon system [26]. The pEYFP::CILK1 plasmid was used as a template, and the YFP-tagged CILK1 gene was subcloned into the pSBbi-Hyg plasmid using the following primers:

pSB_CILK1YFP_forward (SfiI) ggcctctgaggccggtttagtgaaccgtcagatcc

pSB_CILK1YFP_reverse (SfiI) ggcctgacaggccgcaagtaaaacctctacaaatg

### Transfection

The patient and control fibroblast cells were transfected with the pSB::CILK1-YFP plasmid with SB100X [27] using a Neon electroporation system (1319 V, 23 ms pulse width, two pulses). Forty-eight hours after transfection, the cells were treated with 500 μg/ml hygromycin for 2 weeks. The pSB::CILK1-YFP expressing stable cell lines were then stored in liquid nitrogen.

### ICC

Sterile, 24 mm poly-D-lysine-coated glass coverslips were used for cell imaging. The cells were washed twice with PBS (137 mM NaCl, 1.8 mM KH2PO4, 10 mM Na2HPO4, and 2.6 mM KCl) and fixed in 4% paraformaldehyde–4% sucrose in PBS for 10 min. The cells were washed thrice with PBS and treated for 5 min with 0.2% Triton X-100 for permeabilization [28]. Cells were treated for one hour with 4% normal donkey serum for blocking, one hour with a primary antibody, and one hour with a secondary antibody (Alexa Fluor). During the final washing stage, 1 μg/ml DAPI was used to stain the nuclei. Fluoromount G was used as the mounting reagent.

### Microscopy

Fluorescence and immunofluorescence microscopy were performed as described by Cevik et al. [29].

## Results

### Clinical and demographic features

**Fig. 1:**
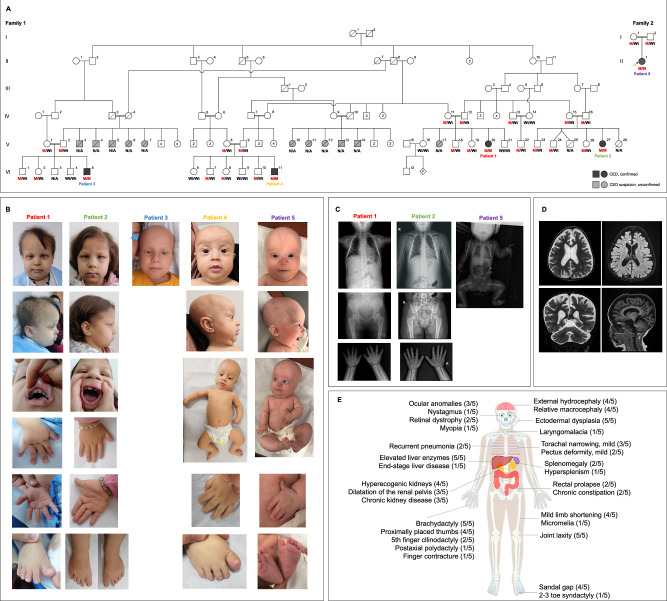
Pedigrees, clinical images, radiographs, and an illustration of the clinical features of the affected individuals. **A** Pedigrees of the patients. The pedigree of Family 1 includes four molecularly confirmed patients with CED (Patients 1–4) and 11 unconfirmed patients suspected of having CED. The pedigree of Family 2 includes Patient 5. M: Mutant, WT: Wild-type, N/A: Not available. **B** Clinical images of the patients. All patients exhibited dolichocephaly, a depressed nasal bridge, full cheeks, ectodermal dysplasia, brachydactyly, and a sandal gap. Written informed consent was obtained from the parents of patients concerning publishing the clinical report along with their clinical pictures. **C** X-ray radiographs of Patients 1, 2, and 5 show a narrow thorax and brachydactyly. Pelvic bones were normal (middle panel). Patient 5 presented with shortening of both proximal and distal parts of the limbs. **D** Brain MRI of Patient 1 at 3 years of age, revealing external hydrocephalus, ventriculomegaly, and cerebral and cerebellar atrophy. **E** An illustration depicting the clinical findings of the patients.

Five patients with similar multisystem syndromic phenotypes were found to have the homozygous c.1664_1665delAT p.(Tyr555Cysfs*48) variant in the *CILK1* gene. All were of Iraqi Turkmen origin, specifically from Tal Afar city in the Nineveh Governorate of northwestern Iraq, and their families had been residing in Türkiye for nearly 10 years. A six-generation pedigree was generated for Family 1, showing that four patients (Patients 1–4) were closely related (Fig. 1A), while Patient 5 belonged to Family 2. Clinical characteristics and images are summarized in Table 1 and Fig. 1B–D, with detailed case reports in the Supplementary Information (see Supplementary Material).

**Table 1:** Clinical and demographic features of the patients.

Patient	Patient 1	Patient 2	Patient 3	Patient 4	Patient 5
**Sex**	Female	Female	Male	Male	Female
**Current age**	7 years	10 years	Died at 9 years	4 months	34 months
**Age at first admission**	1 year	1 year	4 years	Neonatal period	Prenatal period
**First sign/symptom**	Polyuria and polydipsia	Rectal prolapse	Hepatomegaly and elevated liver enzyme levels	Cholestasis	IUGR and limb shortening
**Prenatal finding**	NA	NA	NA	Cystic appearance in the liver	IUGR and limb shortening
**LGA at birth**	−	NA	NA	−	−
**SGA at birth**	−	NA	NA	−	+
**Macrocephaly at birth (including relative)**	NA	NA	NA	+	+
**Short stature**	+	+	+	+	+
**Low weight**	−	−	+	+	+
**Macrocephaly (including relative)**	+	+	+	−	+
**Dolichocephaly**	+	+	−	+	+
**Craniosynostosis/cranial surgery**	−	−	−	−	−
**High forehead**	+	+	+	−	+
**Hypertelorism**	−	−	+	+	+
**Nystagmus**	+	−	−	−	−
**Retinal dystrophy**	+	−	+	−	−
**Myopia**	−	+	−	−	−
**Broad nasal bridge**	+	+	+	+	+
**Low-set or dysplastic, or prominent ears**	−	−	−	−	+
**Full cheeks**	+	+	+	+	+
**Narrow chest/short ribs/pectus deformity**	+	+	−	−	+
**Joint laxity**	+	+	+	+	+
**Mild limb shortening**	+	+	+	+	−
**Micromelia**	−	−	−	−	+
**Brachydactyly of fingers and toes**	+	+	+	+	+
**Clinodactyly**	+	+	−	−	−
**Sandal gap**	+	+	+	−	+
**Finger contracture**	−	−	−	−	+
**Postaxial polydactyly**	−	−	−	−	+
**Proximally placed thumb**	+	+	−	+	+
**Syndactyly (mild, 2**–**3 toes)**	−	−	−	+	−
**Dental abnormalities**	+	+	+	NA	Delayed eruption
**Nail abnormalities**	+	+	+	−	−
**Thin and/or sparse hair**	+	+	+	+	+
**Nevus flammeus/hemangioma**	−	−	−	+	+
**Skin laxity**	+	+	NA	+	+
**Umbilical hernia**	+	−	−	−	−
**Renal cysts**	+	−	−	−	−
**Increased kidney echogenicity**	+	+	+	−	+
**Dilatation of the renal pelvicalyceal system**	+	−	−	+	+
**Chronic renal failure/nephronophthisis**	+	+	+	−	−
**Renal replacement therapy**	+	+	−	−	−
**Elevated liver enzymes**	+	+	+	+	+
**Hepatomegaly**	−	−	+	−	−
**Splenomegaly**	−	+	+	−	−
**Accessory spleen**	+	−	−	−	−
**End-stage liver disease**	−	−	+	−	−
**Congenital cardiac anomaly**	−	−	−	+	+
**Left ventricular hyperplasia**	+	+	+	−	−
**Recurrent respiratory infections**	+	−	−	−	+
**Laryngomalacia**	−	−	−	−	+
**Constipation**	+	+	−	−	−
**Rectal prolapse**	+	+	−	−	−
**Feeding problems**	−	−	−	−	+
**Developmental delay (predominantly motor delay)**	+	+	+	−	+
**Enlarged extracerebral fluid spaces/ventriculomegaly**	+	+	+	−	+
**Cerebral atrophy**	+	+	−	−	+
**Cerebellar atrophy**	+	+	−	−	−
**Brainstem anomaly**	−	−	−	−	+
**Absence of septum pellucidum**	−	−	+	−	−
**Delayed white matter myelination**	−	−	−	−	+
**Anemia**	+	+	+	−	−
**Cytopenia (multiple)**	−	−	+	−	−

*LGA* Large for gestational age, *NA* Not available, *SGA* Small for gestational age.

The patient group included three females and two males. Patients 1, 2, 4, and 5 were alive at 7, 10, 0.44, and 3 years, respectively. Patient 3 died of chronic liver disease at age 9. All patients had disproportionately short stature with skeletal involvement, ectodermal dysplasia, liver involvement ranging from elevated liver enzyme levels to cirrhosis, and kidney involvement ranging from hyperechogenic kidneys or dilatation of the renal pelvis to end-stage chronic kidney disease; four of them had relative macrocephaly associated with external hydrocephaly (Fig. 1E). A clinical diagnosis of CED was made based on shared clinical features, although there was some variability in the disease course and severity among the patients. Family histories suggested at least 11 additional suspected but unconfirmed cases in Family 1 (Fig. 1A). The majority of these individuals were born and died between the ages of 1 and 13 years in Iraq, one to two decades ago, and their medical records or biological materials were not accessible. Based solely on the medical histories provided by their families, these individuals were considered to have clinical features consistent with those of CED. However, we did not include their clinical characteristics in Tables 1 and 2 because of their unconfirmed status.

**Table 2:** Clinical comparison of *CILK1*-related disorders and cranioectodermal dysplasia.

Disease name	*CILK1*-related Endocrine cerebroosteodysplasia	*CILK1*-related Short-rib-polydactyly syndrome	Cranioectodermal dysplasia^a^	*CILK1*-related Cranioectodermal dysplasia (this study)
**Gene**	*CILK1*	*CILK1*	*WDR35, IFT22, IFT140, WDR19, IFT43*	*CILK1*
**MIM gene number**	612325	612325	613602, 606045, 614620, 608151, 614068	612325
**MIM phenotype number**	612651	−	613610, 218330 - 614378, 614099	−
**Mode of transmission**	AR	AR	AR	AR
**Number of families (with molecular diagnosis)**	2	1	38^b^	2
**Number of patients (with molecular diagnosis)**	7	1	50^b^	5
**Sex**	2 F, 5 M	NA	17 F, 32 M, 1 NA^b^	3 F, 2 M
**Perinatal death**	100% (7/7)	+	1 report	0% (0/5)
**Dead at childhood**	0% (0/7)	−	15% (10/65)	20% (1/5)
**Short stature**	0% (0/7)^c^	+^c^	>75%	80% (4/5)
**Macrocephaly (including relative)**	67% (4/6)^c^	+^c^	53%^d^	80% (4/5)
**Dolichocephaly**	42% (3/7)	−	>75%	80% (4/5)
**Retinal dystrophy**	33% (1/3)	NA	<25%	40% (2/5)
**Microphthalmia**	100% (7/7)	NA	NR	0% (0/5)
**Single nostril**	50% (1/2)	−	NR	0% (0/5)
**Presence of premaxilla**	42% (3/7)	−	NR	0% (0/5)
**Cleft palate**	100% (7/7)	−	2 reports	0% (0/5)
**Cleft lip**	100% (7/7)	−	NR	0% (0/5)
**Narrow chest/short ribs/pectus deformity**	28% (2/7)	+	50%–75%	60% (3/5)
**Limb shortening (severe or micromelia)**	100% (7/7)	+	2 reports	20% (1/5)
**Limb shortening (mild or rhizomelic)**	0% (0/7)	−	>75%	80% (4/5)
**Hip dysplasia**	100% (7/7)	+	<25%	0% (0/5)
**Brachydactyly**	100% (7/7)	NA	>75%	100% (5/5)
**Syndactyly (mild, 2**–**3 toes)**	0% (0/7)	−	25%–50%	20% (1/5)
**Syndactyly (severe)**	100% (7/7)	−	3 reports	0% (0/5)
**Polydactyly**	100% (7/7)	+	25%–50%	20% (1/5)
**Joint laxity**	NA	NA	25%–50%	100% (5/5)
**Sparse &/or thin hair**	NA	NA	50%–75%	100% (5/5)
**Dental abnormalities**	NA	NA	50%–75%	100% (4/4)
**Abnormal nails**	NA	NA	25%–50%	60% (3/5)
**Skin laxity**	67% (4/6)^e^	NR	25%–50%	100% (4/4)
**Bilateral inguinal hernias**	NR	NR	25%–50%	0% (0/5)
**Nephronophthisis/chronic kidney disease**	NA	NA	50%–75%	60% (3/5)
**Increased kidney echogenicity**	57% (4/7)	−	50%–75%	80% (4/5)
**External genitalia abnormalities**	71% (5/7)	+	2 reports	0% (0/5)
**Liver disease (hepatic fibrosis, cirrhosis, &/or hepatomegaly)**	NA	NA	25%–50%	40% (2/5)
**Congenital cardiac anomalies**	25% (1/4)	NR	<25%	40% (2/5)
**Recurrent lung infections**	NA	NA	25%–50%	40% (2/5)
**Developmental delay (most often motor development)**	NA	NA	50%–75%	80% (4/5)
**Intellectual disability**	NA	NA	<25%	0% (0/3)
**Enlarged extracerebral fluid spaces/ventriculomegaly**	100% (3/3)	+	44%^f^	80% (4/5)
**Holoprosencephaly**	80% (4/5)	−	NR	0% (0/5)
**Corpus callosum anomaly**	100% (4/4)	NA	NR	0% (0/5)
**Endocrine hypoplasia**	100% (3/3)	NA	NR	0% (0/5)
**Cystic hygroma**	14% (1/7)	−	<25%	0% (0/2)

*AR* autosomal recessive, *F* female, *M* male, *NA* not available or not applicable, *NR* not reported, *+* Present, *−* Absent.

^a^The statistical data is taken from the internet textbook Genereviews.

^b^Includes the latest reports published after the latest version of the Genereviews internet textbook.

^c^According to the birth parameters.

^d^Macrocephaly was found in 8 of 15 patients for whom growth parameters were reported.

^e^Excess skin below the chin.

^f^External hydrocephalus or ventriculomegaly was found in 8 of 18 patients with available autopsy or cranial imaging.

### A homozygous *CILK1* variant causes Cranioectodermal dysplasia (CED)

**Fig. 2:**
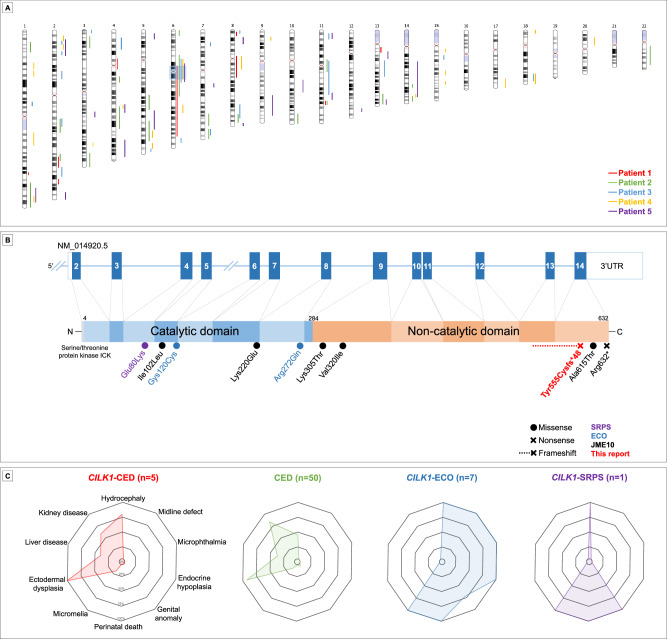
Identification of the causal variant in *CILK1* in individuals with CED phenotype. **A** Homozygosity stretch identification showed only a single locus shared among the patients, chr6:51732807-71011831. The colored bars indicate regions of homozygosity found in each patient. The shaded box indicates the homozygosity stretch region. **B** Schematic of the localization of genotypes associated with CILK1-related phenotypes on the transcript and protein. The variant reported in this study is located on a quite distant part of the protein, in contrast to ECO and SRPS-related variants. **C** The diagram shows the frequency of cardinal features in *CILK1*-related ciliopathies, CED, and the patients in this study. Note that the novel *CILK1*-related phenotype closely resembles CED. The patients in this report are labeled as *CILK1*-CED and represented in red.

Clinical ES analysis did not detect pathogenic variants in known CED-related genes, suggesting a causal variant in another gene. In homozygosity mapping of the ES data of the patients, the only region of homozygosity shared among the patients was approximately 19 Mb in length, chr6:51,732,807–71,011,831 (Fig. 2A). The analysis of the ES data focused on the homozygosity stretch region only showed the shared homozygous frameshift variant in the *CILK1* gene: NM_014920.5; c.1664_1665delAT; p.(Tyr555Cysfs*48); chr6:52871191:CAT>C (Fig. S2). No additional rare disruptive variants were found in the ES reanalysis. The homozygous frameshift variant c.1664_1665delAT, p.Tyr555Cysfs*48 is referred to as the *CILK1* variant henceforth.

*CILK1* (also known as Intestinal Cell Kinase, *ICK*) consists of 14 exons and encodes a serine/threonine kinase protein with a length of 632 amino acids. The c.1664_1665delAT deletion occurred in exon 13. It substitutes tyrosine with cysteine at position 555 and causes a frameshift and a premature termination codon (stop codon) at amino acid position 602 of the CILK1 protein. (Fig. 2B). Since the variant introduces a stop codon in the last exon, it is predicted to escape nonsense-mediated mRNA decay (NMD). RT-qPCR on patient-derived fibroblasts confirmed comparable *CILK1* mRNA expression to controls (Fig. S7). These data confirmed that the mutant *CILK1* allele is expressed at the RNA level.

Haplotype analysis across a 40.5 Mb region showed a shared segment ~19 Mb in length, suggesting a founder effect (Table S2). Segregation analysis in available relatives confirmed that only affected individuals were homozygous, while parents were heterozygous and unaffected relatives were heterozygous or wild-type (Fig. 1A, Fig. S3).

Biallelic variants in the *CILK1* gene have been previously implicated in lethal skeletal ciliopathy phenotypes, ECO, and SRPS, and the variants were located in the kinase domain (Fig. 2B). The *CILK1* variant is located in the C-terminal non-catalytic domain and is predicted to cause a truncated protein, which differs in variant type and location from the *CILK1*-associated ciliopathies of ECO and SRPS. Clinical features in our cohort aligned with CED, with more prolonged survival and less severe organ involvement than ECO or SRPS (Fig. 2C, Table 2). Finally, since some heterozygous variants of this gene have been reported to cause EJM10, we reinvestigated all family members for a history of epilepsy. No epileptic phenotypes were observed in heterozygous or homozygous individuals.

### The cilia were elongated in the fibroblast cells of the patient

**Fig. 3:**
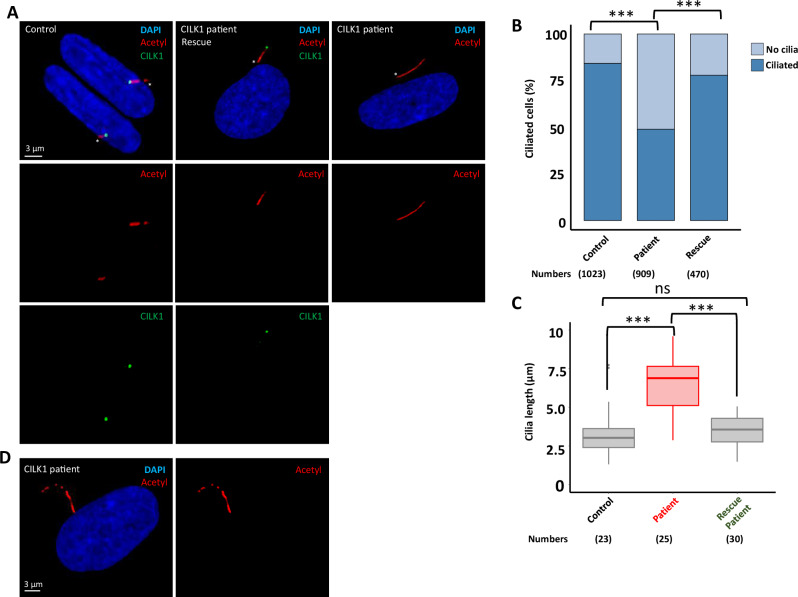
The disease-causing variant in *CILK1* leads to longer cilia in patient-derived cells. **A** Immunofluorescence images of control, patient-derived, and rescue of patient-derived cells. Staining includes DAPI (blue), acetylated tubulin (red), and *CILK1* (green). An asterisk marks the ciliary tip. Scale bar: 3 μm. **B**, **C** Bar and scatter plots show the percentage of ciliated and non-ciliated cells and ciliary lengths after serum deprivation in control, patient-derived, and rescued patient-derived cells. **D** Immunofluorescence images of patient-derived cells displaying curly cilia. Staining includes DAPI (blue) and acetylated tubulin (red). Scale bar: 3 μm.

*CILK1* and its orthologs were already implicated in cilia length determination in various organisms [10, 11, 13, 14, 16, 30]. We therefore evaluated ciliogenesis and ciliary length in the fibroblast cells obtained from a patient (Patient 1) and age-matched healthy controls for comparative analysis. Next, we quantified the number of cells with and without cilia, revealing a subtle reduction in the number of ciliated cells in the patient-derived cells compared to the control cells (Fig. 3A, B). Next, we assessed cilia length in both control and patient-derived cells and observed a two-fold increase in cilia length in patient cells compared to the control (Fig. 3C). We occasionally observed the curvature of elongated cilia in patient-derived cells, as previously documented in *CILK1* knockout cells [17] (Fig. 3D).

Importantly, we introduced the CILK1::YFP construct into control and patient cell lines to establish stable patient cell lines expressing CILK1::YFP. Consistent with previous reports, CILK1::YFP was localized at the ciliary base in control and patient-derived cells (Fig. 3A, Fig. S4A, B). Remarkably, CILK1::YFP successfully rescued both observed ciliary phenotypes in patient cells, including long cilia and reduction in ciliated cell numbers (Fig. 3B, C). The successful rescue of ciliary abnormalities in patient-derived human cell lines strongly suggests that these are attributable to the CILK1 variant, which affects the last part of the C-terminal non-catalytic region in CILK1.

Next, we used CRISPR/Cas9 to insert Green Fluorescent Protein (GFP) into the C-terminal region of the *C. elegans* DYF-5, the *C. elegans* ortholog of CILK1/ICK, creating an endogenous DYF-5::GFP line. To further investigate the conservation of the affected residues, we performed multiple sequence alignments between human CILK1 and *C. elegans* DYF-5 (Fig. S5A). Next, we used ConVarT [31] to assess whether the human disease-causing variant had orthologous counterparts carried by mutant strains in mice or *C. elegans*. After confirming that no such orthologous variant exists, we used CRISPR/Cas9 to introduce the orthologous disease-causing CILK1 variant into endogenous DYF-5::GFP to observe its impact on DYF-5::GFP localization. To monitor cilia, we incorporated OSM-3::mCherry (the human ortholog KIF17 and a ciliary marker) into both endogenous DYF-5::GFP and DYF-5::GFP (p.Tyr555Cysfs*48). Similar to a previous work [32], DYF-5 was enriched at the ciliary tip in both head (amphid) and tail (phasmid) (Fig. S5B). The localization of DYF-5::GFP was unaltered when the CILK1 variant was introduced into DYF-5::GFP, suggesting that alterations in the C-terminal non-catalytic region did not affect ciliary tip localization (Fig. S5B). However, our data are consistent with previous data [17] showing that the last part of the C-terminal non-catalytic region is not required for ciliary localization. Notably, the dye-filling assay in the mutant strain did not reveal any apparent defects in sensory cilia architecture as their ability to take up the lipophilic fluorescent dye was essentially normal, while fluorescently tagged IFT-74 suggested potential abnormalities at the ciliary tips in the mutant background; however, these findings require further confirmation/rescue and are not included in the current manuscript (data not presented).

### Ciliary distributions of IFT proteins were altered in patient-derived fibroblast cells

Mutations affecting *CILK1* have previously been demonstrated to extend cilia length and lead to the accumulation of ciliary proteins at the cilia tip. Therefore, we examined the localization and staining patterns of ARL13B small GTPase, polyglutamylation, and CEP164 (a crucial basal body component). Staining of all these markers with their corresponding antibodies revealed that none of their localizations/staining was altered in patient-derived fibroblast cells (Fig. S6).

**Fig. 4:**
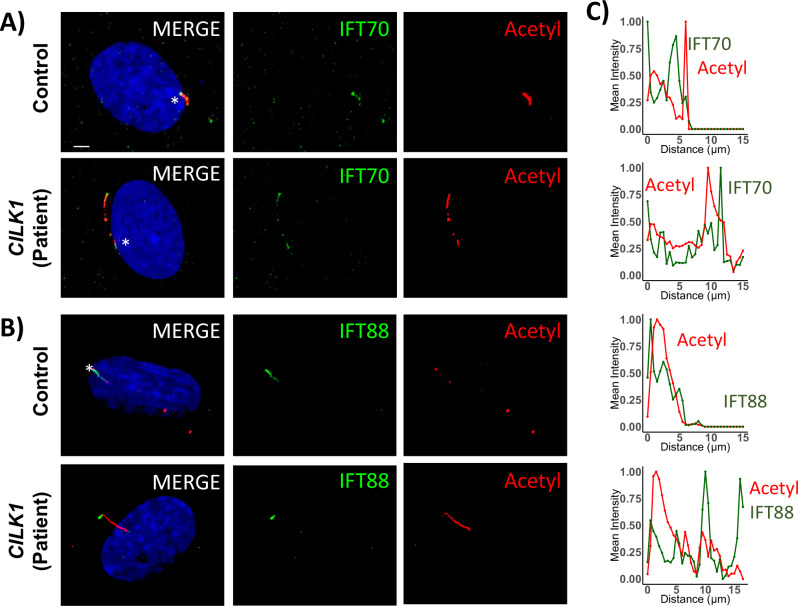
IFT distribution is altered in patient-derived cells. **A**, **B** Immunofluorescence images of control and patient-derived cells showing staining for acetylated tubulin (red), IFT88 (green), IFT70 (green), and DAPI (blue). Scale bar: 5 μm. **C** Density plot illustrating the distribution of acetylated tubulin (red), IFT88 (green), and IFT70 (green) along the ciliary length.

Next, we performed dual staining on control and patient-derived cells using acetylated tubulin in conjunction with either IFT70 or IFT88 antibodies and quantified their fluorescence intensity across the entire cilium (Fig. 4A, B). Both IFT88 and IFT70 are components of the IFT-B complex, and antibodies against these proteins exhibited intense staining at the ciliary base in control cells. The localization patterns of IFT components, including IFT88 and IFT70, appeared to be affected in patient-derived fibroblast cells, with apparent accumulation-like staining in the ciliary tip and punctate-like patterns across the cilia (Fig. 4C).

## Discussion

Data from ES analysis, patient-derived cells, and in vivo models demonstrate that the homozygous frameshift variant (c.1664_1665delAT, p.Tyr555Cysfs*48) in *CILK1* causes CED by reducing cilia number, increasing cilia length, and altering the localization of IFT components. While *CILK1* has previously been associated with lethal skeletal ciliopathies, our findings demonstrate that variants in *CILK1* can also underlie a milder, childhood-onset phenotype consistent with CED (Fig. 2C, Table 2). This observation expands both the phenotypic and genotypic spectrum of *CILK1*-associated ciliopathies. Moreover, this discovery challenges the existing paradigm of CED pathogenesis, which has primarily focused on structural components of the IFT machinery, by underscoring the critical role of regulatory kinases such as CILK1 in maintaining ciliary architecture and signaling pathways [33]. These findings expand the genetic and mechanistic landscape of CED, support the inclusion of *CILK1* in diagnostic gene panels even for atypical presentations, and highlight its potential as a therapeutic target.

The variant identified in our patients introduces a premature termination codon in the last exon, truncating the final 30 amino acids within the intrinsically disordered, non-catalytic C-terminal domain. Previous studies on CILK1 variants located in the C-terminal domain (e.g., p.Lys305Thr, p.Val320Ile, p.Ala615Thr, and p.Arg632*) retained the ability to phosphorylate KIF3A at Thr672 with an efficiency comparable to wild-type CILK1 [34]. Further supporting that kinase activity may be maintained, Nakamura et al. investigated a series of C-terminal truncation constructs and a kinase-dead mutant. They found that while partial C-terminal truncations partially rescued ciliary defects, the kinase-dead mutant failed to do so despite its proper localization [13]. This indicates that CILK1’s kinase function is necessary, but not solely sufficient, implying that the C-terminal domain contributes to an additional role, such as interaction with IFT components, which may be disrupted in our variant. Although we have not yet performed kinase assays specifically for the CILK1 variant, the functional parallels with Arg632* and the broader mutational analyses support the interpretation that kinase function is likely preserved. In contrast, other C-terminal-dependent functions may be compromised.

Findings from our *C. elegans* model, where the DYF-5 ortholog of CILK1 retained proper localization but exhibited elongated cilia and impaired retrograde transport, support a loss-of-function mechanism affecting IFT dynamics [11, 35]. In parallel, fibroblasts derived from a patient showed a reduced ciliation rate, increased cilia length, and accumulation of IFT proteins at the ciliary tip, consistent with defects in retrograde trafficking. These findings are aligned with phenotypes observed in other forms of CED caused by mutations in IFT-A genes [2, 5–8]. However, a striking difference lies in cilia length: while CED caused by *WDR35*, *IFT43*, or *WDR19* is associated with shortened cilia (~2.2–2.9 µm) [36], *CILK1*-related CED is associated with elongated cilia (~4.6 µm). This phenotype resembles asphyxiating thoracic dystrophy (ATD) caused by *WDR19* mutations, which result in longer cilia (~4.73 µm in ATD patients). Similarly, *DYNC2H1* causes longer cilia (~4.7–5.0 µm in ATD patients) but shorter cilia in severe Short-rib-polydactyly syndrome (SRPS; ~2.3 µm) [36]. These observations suggest that CILK1 acts upstream of the IFT system to regulate cilia length. Thus, the phenotypic differences raise questions about the current classification systems based on ciliary morphology [36]. The framework proposed by Doornbos et al., which categorizes ciliopathies according to three parameters, including the cilia length, may not adequately accommodate *CILK1*-related CED. This highlights the need for ongoing refinement of scoring systems as new findings emerge. Despite morphological differences, defective retrograde IFT appears to be the shared pathogenic mechanism across CED subtypes. Further studies comparing CILK1-mutant fibroblasts with those from patients with IFT-A-related CED are warranted to clarify whether *CILK1* mutations define a distinct mechanistic subtype. To explore whether *CILK1* defines a distinct CED subtype, introducing *CILK1* and *IFT* mutations (e.g., *IFT52*, *IFT122*) into animal models like zebrafish could compare double versus single mutants, revealing shared versus unique IFT defects. While such a comparison was not feasible in this study, it remains an important future direction.

Clinically, all five patients harboring the *CILK1* variant had a relatively normal postnatal period, with developmental features of CED emerging over time. As seen in other forms of CED, disease progression involved renal and hepatic deterioration, ultimately resulting in childhood mortality in some cases [1]. The severity and combination of affected organs varied between individuals. While some patients had more pronounced skeletal findings, others exhibited earlier or more severe hepatic involvement. We did not identify a precise genotype–phenotype correlation within this cohort, but this variability is consistent with that seen in other ciliopathies [1, 3, 4]. Brain imaging of four patients identified intracranial abnormalities, including external hydrocephalus, and additional findings. Although only a subset of previously reported cases have undergone neuroimaging or autopsy, hydrocephalus and other intracranial anomalies have been documented [1]. In addition to the core features of CED, we identified several findings that were not commonly reported in the literature. These included rectal prolapse, chronic constipation, and accessory spleen. While these features are not defining for CED, they have been occasionally reported in isolated cases and may represent part of the broader disease spectrum. The accessory spleen, observed in Patient 1, has only been documented previously [37]. Interestingly, another patient in the literature presented with a situs anomaly [38]. The rare occurrence of both findings suggests that lateralization defects may represent an additional feature of CED. While rectal prolapse has not been previously reported in CED, connective tissue abnormalities, such as joint and skin laxity, are well-documented in nearly half of the cases [1]. These findings suggest that connective tissue involvement may underlie the novel features of rectal prolapse in CED patients.

To date, at least 50 patients from 38 families with a molecular diagnosis of CED have been described in the literature [1, 39–47]. Most cases were described in small series or as isolated reports, often without long-term follow-up. The review by Lin et al. in 2013 summarized only 24 molecularly diagnosed patients [47], and more recent compilations (e.g., GeneReviews) also reflect limited data [1]. The patients presented here add valuable clinical and molecular information to the CED phenotype, particularly regarding early neurologic imaging, organ involvement trajectory, and non-canonical features.

When considering other types of CED, a key observation is that nearly all genes implicated in its etiology, including *CILK1*, are associated with multiple ciliopathy phenotypes. Notably, all genes except *IFT122* have been linked to both lethal and non-lethal skeletal ciliopathy phenotypes, which exhibit significant phenotypic overlap with CED. Interestingly, a few reports of affected individuals from the same family presenting with different phenotypes in this disease group, characterized by high allelic and clinical heterogeneity [44]. Moreover, hypomorphic or missense mutations in *IFT140* may cause milder, tissue-specific conditions, such as retinitis pigmentosa or isolated renal cysts [48, 49]. In most patients with CED, at least one variant found in genes encoding IFT-A subunits is missense, and it is assumed that biallelic loss-of-function variants in these genes are incompatible with life. Our findings support this model as the p.Tyr555Cysfs*48 variant affects the non-catalytic domain, and the milder phenotype observed in our patients is consistent with residual protein function. This aligns with allelic heterogeneity observed in other ciliopathies, where mutation type and location influence phenotypic severity.

Clinical variability in *CILK1*-related CED, with differing liver or skeletal predominance, likely reflects genetic modifiers (e.g., *IFT122, IFT52, GLI3*) or environmental factors, although no explicit pathogenic modifiers were identified in our cohort. Animal models introducing *CILK1* and *IFT* mutations and polygenic risk score analyses could elucidate modifier and environmental contributions, refining CED’s diagnostic and therapeutic approaches [32, 33].

The variant described here introduces a premature stop codon in the final exon of *CILK1* and is thus predicted to escape NMD. Recent findings suggest that NMD-escaping variants can cause dominant disease, particularly in neurodevelopmental disorders [50, 51]. However, none of the 22 heterozygous carriers in our families exhibited clinical symptoms such as epilepsy or developmental delay. Furthermore, the variant is absent from population databases, and *CILK1* has a pLI score of 0 and a LOEUF score of 0.774, suggesting tolerance to heterozygous pLoF variation. These data support a recessive inheritance model with low risk for dominant-negative or gain-of-function effects. Initially, EJM10 was attributed to *CILK1* haploinsufficiency; however, heterozygous null or ECO-associated p.Arg272Gln variants in mice did not result in epilepsy [20, 52]. Moreover, recent studies modeling the EJM10-associated p.Ala615Thr variant in the non-catalytic region demonstrated increased ciliogenesis and shorter cilia in cellular models, suggesting gain-of-function effects [53]. The absence of epilepsy or neurologic abnormalities among heterozygous carriers in our cohort further supports the idea that the p.Tyr555Cysfs*48 variant is not associated with EJM10 or neurodevelopmental phenotype.

All five affected individuals shared a homozygous haplotype, possibly consistent with a founder mutation in the Iraqi Turkmen population. This has practical implications for genetic counseling and carrier testing in at-risk communities. As with other autosomal recessive disorders, the recurrence risk for siblings is 25%. Community-specific screening and cascade testing should be considered.

In conclusion, we demonstrate that the homozygous frameshift variant in the non-catalytic domain of CILK1 causes cranioectodermal dysplasia through disruption of retrograde IFT and ciliary dynamics. These findings define *CILK1* as a novel causal CED gene and expand the phenotypic and mechanistic landscape. This report also highlights the importance of including upstream regulators, not only structural components, in both diagnostic and mechanistic frameworks for this group of ciliopathies.

## Supplementary information

Supplemental Material

Figure S1

Figure S2

Figure S3

Figure S4

Figure S5

Figure S6

Figure S7

Table S2

## Data Availability

The data supporting the results of this study are available in the article and Supplementary Information, or can be made available to the corresponding authors upon reasonable request. The variant has been submitted to ClinVar and is reachable with the accession number SCV005374869.
